# Draft Genome Sequence of the Novel Exopolysaccharide-Producing Bacterium *Altibacter lentus* Strain JLT2010^T^, Isolated from Deep Seawater of the South China Sea

**DOI:** 10.1128/genomeA.00954-14

**Published:** 2014-10-23

**Authors:** Zilian Zhang, Yi Chen, Yingnan Fu, Nianzhi Jiao

**Affiliations:** State Key Laboratory of Marine Environmental Science, Institute of Marine Microbes and Ecospheres, Xiamen University, Xiamen, People’s Republic of China

## Abstract

*Altibacter lentus* strain JLT2010^T^ is the type strain of the recently identified novel genus and species of the family *Flavobacteriaceae* and was first isolated from deep seawater of the South China Sea. It can produce exopolysaccharide. Here we report the first draft genome of JLT2010^T^ (3,160,033 bp, with GC content of 42.12%) and major findings from its annotation. It is the first reported genome in the genus *Altibacter*.

## GENOME ANNOUNCEMENT

*Altibacter lentus* JLT2010^T^ (JCM 18884^T^; CGMCC 1.12167^T^), the type strain of *Altibacter lentus* gen. nov., sp. nov., was originally isolated from deep seawater at 2,000 m depth of the South China Sea (20°N, 118°E) (1). This strain is chemoheterotrophic, aerobic, Gram-negative, rod-shaped, and yellow-pigmented, and it forms a distinct phylogenetic lineage within the family *Flavobacteriaceae* (1). The strain can produce exopolysaccharide. Most notably, the members of *Flavobacteriaceae* are especially proficient in degrading various biopolymers such as cellulose, chitin, and pectin (2), and thus have significant contribution to the remineralization of marine organic matter (3). The genome sequencing was performed to study the synthetic and metabolic system of exopolysaccharide as well as other biopolymers such as xylan and chitin in the novel type strain JLT2010^T^. Here, we present the draft genome sequence of strain JLT2010^T^. This is the first genome report of the bacterial genus *Altibacter*.

The genome of JLT2010^T^ was sequenced by a whole-genome shotgun strategy using the Illumina HiSeq 2000 sequencing platform. Genome sequences were assembled *in silico* using SOAPdenovo (4), resulting in 17 contigs with an *N*_50_ length of 1,823,738 bp. Gene prediction was prepared using Glimmer 3.0 software (5). Annotation of the coding sequences (CDSs) was performed by searching against the KEGG (6), COG, Swiss-Prot, NCBI-NR, and GO (7) protein databases. rRNA and tRNA genes were found using rRNAmmer (8) and tRNAscan (9), respectively.

The draft genome included 3,160,033 bases with a GC content of 42.12%. A total of 2,976 genes were predicted, with an average length of 966 bp, accounting for 90.99% of the genome. There are 35 tRNA sequences. The 1,858 genes (62.4%) can be annotated in the KEGG orthology system. A total of 177 genes were related to carbohydrate metabolism in the KEGG orthology system. In addition, the 928 genes can be classified into 20 functional COG categories. Among them, 33 genes encoded proteins involved in carbohydrate transport and metabolism and 54 genes encoded proteins involved in inorganic ion transport and metabolism, suggesting that this novel isolate is capable of utilizing a wide range of carbohydrate sources and is better suited for nutrient limiting environments. The genes encoding the synthesis and secretion of exopolysaccharide and the metabolism of other biopolymers such as xylan and chitin were uniquely identified in the genome sequences of the strain JLT2010^T^.

### Nucleotide sequence accession numbers.

This whole-genome shotgun project has been deposited at DDBJ/EMBL/GenBank under the accession number JPZT00000000. The version described in this paper is version JPZT01000000.
